# Chemically defined polyethylene glycol siRNA conjugates with enhanced gene silencing effect

**DOI:** 10.1016/j.bmc.2014.02.004

**Published:** 2014-04-01

**Authors:** Zuzana Gaziova, Volker Baumann, Anna-Maria Winkler, Johannes Winkler

**Affiliations:** University of Vienna, Department of Medicinal Chemistry, Althanstraße 14, 1090 Vienna, Austria

**Keywords:** Synthetic oligonucleotides, PEGylation, RNA interference, Oligonucleotide conjugates, Gene silencing

## Abstract

The therapeutic application of siRNA suffers from poor bioavailability caused by rapid degradation and elimination. The covalent attachment of PEG is a universal concept to increase molecular size and enhance the pharmacokinetic properties of biomacromolecules. We devised a facile approach for attachment of PEG molecules with a defined molecular weight, and successful purification of the resulting conjugates. We directly conjugated structurally defined PEG chains with twelve ethylene glycol units to the 3′-terminal hydroxyl group of both sense and antisense strands via an aminoalkyl linker. The conjugates were easily purified by HPLC and successful PEGylation and molecule integrity were confirmed by ESI-MS. The evaluation of in vitro gene knockdown of two different targets in MCF-7 breast cancer cells showed stable pharmacologic activity when combined with a standard transfection reagent. Sense strand PEGylation even increased the silencing potency of a CRCX4-siRNA which had modest activity in its wild-type form. The results indicate that PEG chains at the 3′-terminus of both strands of siRNA are well tolerated by the RNAi effector. The attachment of short, chemically defined PEG chains is a feasible approach to improve the pharmacokinetic properties of siRNA, and can be combined with other targeted and untargeted delivery vehicles.

## Graphical abstract

**Figure:**
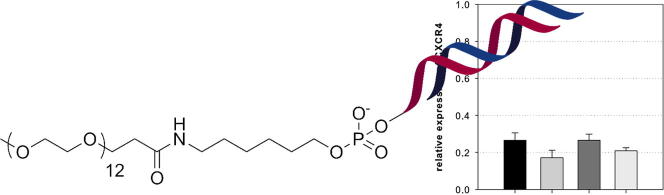

## 1 Introduction

The development of gene silencing oligonucleotides for therapeutic applications has recently seen encouraging results. In early 2013 the first systemically applied antisense compound, mipomersen has won market approval in the USA for the treatment of homozygous familiar hypercholesterolemia.^1^ Positive clinical results of the application of splice-switching agents have been reported for the treatment of Duchenne muscular dystrophy (DMD). Two distinct oligonucleotides, eteplirsen^2^ and drisapersen^3^ have resulted in restoration of functional dystrophin protein in human patients, and improved the clinical outcome. Mipomersen is a second generation phosphorothioate antisense oligonucleotide with terminal 2′-*O*-methoxyethyl nucleosides, drisapersen is a phosphorothioate with flanking 2′-*O*-methoxy nucleosides, and eteplirsen is a phosphorodiamidate morpholino oligomer (PMO).

siRNA oligonucleotides have not yet been evaluated in comprehensive clinical trials, but preclinical development has shown encouraging results. Because chemical optimization is poorly tolerated by the molecular effectors, the main attention has been shifted to focus on the development of delivery systems. Lipid nanoparticles (SNALPs) have been evolved to increase delivery efficiency and have enabled a significant lowering of the dose necessary for intracellular gene silencing.^4,5^ Other delivery systems have been designed for decomposition after successful cellular uptake, enhancing release of the siRNA cargo and facilitating endosomal escape.^6,7^ Using disulfide and acid labile bonds, targeting ligands *N*-acetyl glucosamine (GalNAc) and masking groups are cleaved from the carrier polymer in the endsome, resulting in a positively charged compound believed to destabilize endosomes through the proton sponge effect.^8,9^

Despite these progresses, several important challenges remain before gene silencing oligonucleotides can be applied in a widespread manner in the therapeutic setting. The example of mipomersen shows that the toxicity issue is far from resolved. Mipomersen has not been granted approval in Europe due to concerns about hepatotoxicity and cardiovascular adverse effects. In the USA, a risk management and mitigation strategy (REMS) has been required by the FDA to gather information about adverse effects in long-term treatment regimens. The phosphorothioate backbone of antisense oligonucleotides not only mediates plasma protein binding and triggers a cellular uptake mechanism, but is also responsible for sequence-unspecific effects, including apoptosis induction and endoplasmatic reticulum stress.^10–14^ Delivery systems for siRNA are so far only effective for hepatic gene silencing, because both lipid nanoparticles and GalNAc conjugated polymers show a high preference for liver tissue.^15^ At present, no strategy for effective siRNA delivery to other tissues is available.

A wide variety of oligonucleotide conjugates with ligands for enhancing bioavailability or adding active targeting have been prepared and evaluated.^16^ Examples include conjugations with small lipophilic molecules (cholesterol, fatty acids, etc.), saccharides such as GalNac, or receptor-targeted peptides and proteins. Additionally, the attachment of short and long polyethylene glycol chains has been investigated for the ability to increase the apparent molecular size of oligonucleotides and alleviate their high hydrophilicity with the aim to increase bioavailability and cell membrane permeation. PEGylation of nucleic acids can most conveniently be done at the 3′- or 5′-OH-group, directly or by introduction of a spacer. It was discovered that this modification extended plasma stability and reduced renal filtration rates.^17^ An antisense oligonucleotide conjugate with PEG 5000 was effective for in vitro and in vivo gene silencing when applied with a polyethylene imine carrier.^18^ A PEGylated aptamer, pegaptanib, was approved by FDA in 2004 for the treatment of age-dependent maculadegeneration (AMD). Here, a branched PEG of 40 kDa is attached to the VEGF-targeted aptamer through a penta-amino linker.

For siRNA, PEG chains with molecular weights between 2 and 20 kDa have been attached to the oligonucleotides.^19–21^ PEGylation generally reduces the gene silencing activity significantly when attached in a non-cleavable manner,^20,21^ but enhances the pharmacokinetic properties. siRNA PEGylation is usually afforded with polydisperse long-chain PEG mixtures, which yields mixtures of conjugates with different PEG chain lengths, complicating purification and analyses. To avoid those problems, we conjugated short PEG chains with defined length and size to siRNA sense and antisense strands. We hypothesized that the molecular components of the RNAi mechanism show better tolerance for shorter PEG chains then for larger ligands, and by using the 3′-hydroxy group of the sense or antisense strand, inhibition of strand recognition was aimed to be minimized. We generated a conjugated oligonucleotide with a defined structure, and show that short PEGyl chains are well tolerated by the molecular siRNA effectors with no decrease in knockdown efficiency.

## 2 Results

**Figure 1:**
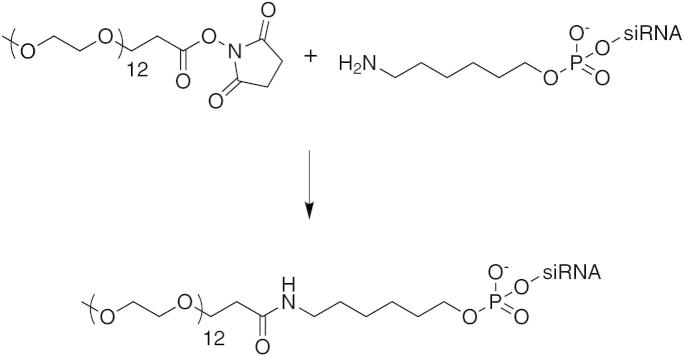
Reaction scheme of NHS–PEG conjugation. The *N*-hydroxy succinimide active ester of a monodisperse PEG with 12 ethylene glycol units was covalently attached to the 3′-aminohexyl linker of a single stranded siRNA oligonucleotide. The reaction was conducted in borate buffer, pH 8.0, with a 25-fold surplus of PEG–NHS–ester. After 1 h, the conversion was nearly complete, and isolated yields were over 75%.

**Figure 2:**
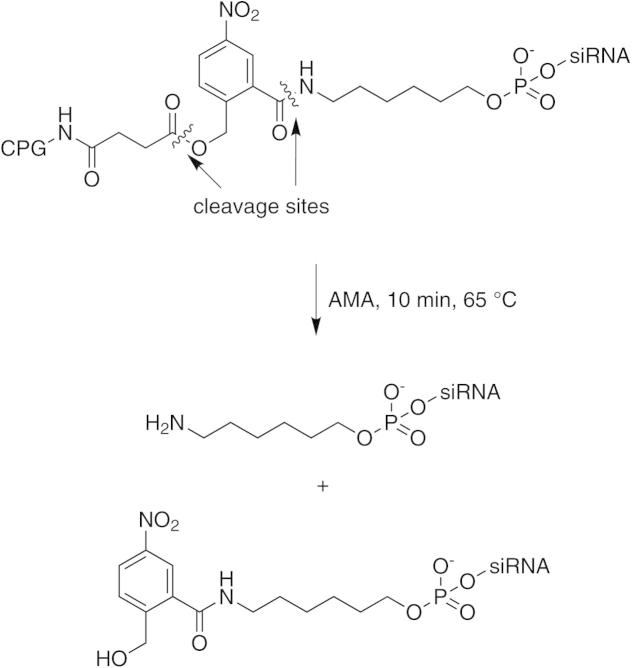
Side product after incomplete deprotection of amino-on CPG. The recommended reaction time for oligonucleotide resin cleavage of 10 min in AMA at 65 °C proved to be insufficient for complete deprotection of the amino function of the amino-on CPG. The nitrophenyl amide was found to be still partially attached. A second deprotection step of the mixture in concentrated ammonia for 4 h provided a homogenous solution of the oligonucleotide with free primary amine. Alternatively the amino-on CPG can be completely deprotected by longer incubation in AMA.

**Figure 3:**
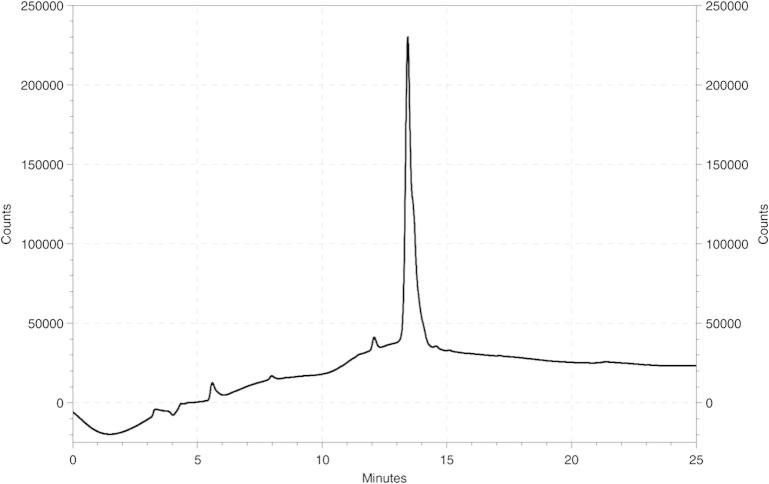
HPLC of PEGylated antisense strand (**5**) after purification. Semi-preparative reversed phase HPLC with a linear gradient of acetonitrile (8–30% in 30 min) in TEAA buffer was used for separation of PEGylated siRNA oligonucleotides **5** and **6** from unreacted educts (**3** and **4**) and NHS-PEG_12_. Analytical HPLC of the respective fractions with the same conditions confirmed the successful purification and high purity of the isolated compound.

The reaction of an *N*-hydroxy succinimide (NHS) activated PEG esters with primary amino groups is an attractive and facile option for the preparation of PEG-conjugated siRNAs (Fig. 1). We used amino-on CPG,^22^ a modified resin for oligonucleotide synthesis, to introduce an aminohexyl function to the 3′-terminus of both antisense and sense strand of the siRNA. After employing the recommended conditions for oligonucleotide cleavage and deprotection (AMA, 10 min at 65 °C), we initially found two different compounds after purification, which were well separated on a RP-HPLC column, but co-migrated in gel electrophoresis. Mass spectrometric analysis revealed that the encountered impurity resulted from incomplete deprotection of the amino-on resin^22^ (Fig. 2). The more labile ester linkage is completely hydrolyzed under these basic conditions, while the amide masking the aminohexyl linker is less reactive, and a mixture of the desired amino functionalized oligonucleotide and the corresponding 2-(hydroxymethyl)-5-nitrobenzamide derivative was isolated. Longer deprotection with concentrated ammonia or AMA afforded the completely deprotected 3′-aminohexyl oligonucleotide.

(PEG)_12_–NHS ester was reacted in an aqueous buffer solution to the aminohexyl linker in a 25-fold molar surplus, yielding conversion rates of over 75%. Monodisperse PEG chains were successfully attached to both the sense and antisense strand of a bcl-2 targeted siRNA. After HPLC purification (Fig. 3) and desalting, the conjugate structure was verified by ESI-MS (Table 1).

**Figure 4:**
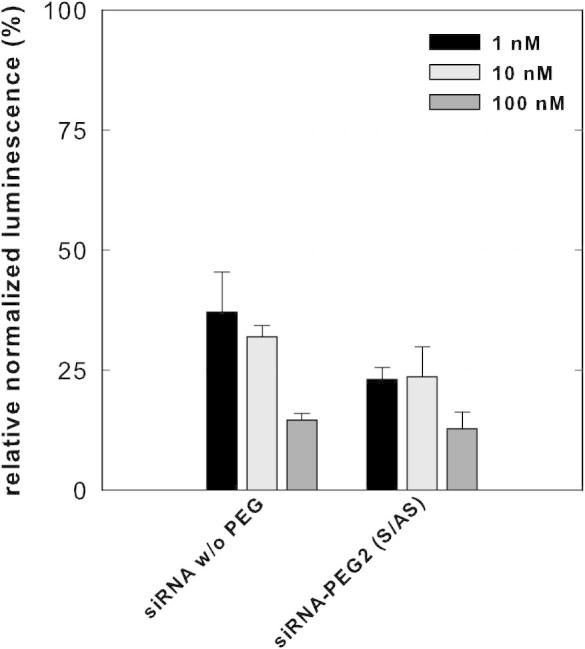
Gene knockdown in a luciferase reporter assay after co-transfection of psiCHECK2-bcl-2 plasmid and siRNA. A psiCHECK2 plasmid with the cDNA of the human bcl-2 gene fused to the Renilla luciferase gene was transfected together with the indicated siRNA into MCF-7 breast cancer cells. Bcl-2 targeted gene knockdown reduces the signal of the Renilla luciferase, while the Firefly luciferase is used for normalization of transfection efficiency and cell number. Knockdown effects are reported in normalized Renilla luciferase intensities (*n* = 3), error bars represent the standard deviation.

The siRNA conjugates were transfected into mammalian MCF-7 cells and the knockdown of the target bcl-2 mRNA was initially investigated with a luciferase reporter assay. We inserted the cDNA of the human bcl-2 gene into the plasmid by molecular cloning, affording a quantitative readout for gene silencing activity (Renilla) with normalization to the second luciferase gene (Firefly). The plasmid was co-transfected with the respective siRNA, and Renilla and Firefly luciferase levels were detected after addition of the appropriate substrates. The siRNA PEGylated on both strands (**5**+**6**) resulted in strong gene silencing (Fig. 4), with potent activity of over 70% already in a nanomolar concentration, and a reduction of nearly 90% of the reporter gene after treatment with 100 nM. Compared to the unmodified sequence (**1**+**2**), the extent of bcl-2 knockdown was not reduced by PEGylation.

**Figure 5:**
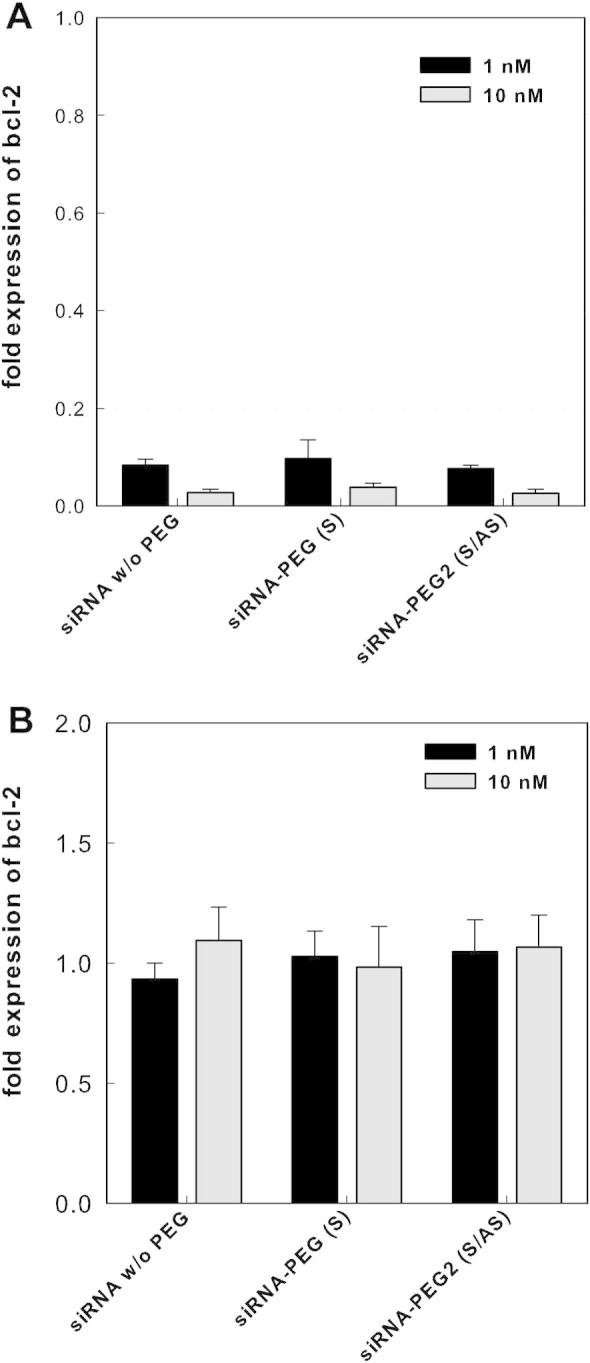
Gene knockdown of endogenous bcl-2. (A) After lipofectamine 2000-mediated transfection, (B) after unassisted application. The effect of PEG-siRNA conjugates (siRNA-PEG (S), **1**/**6**; siRNA-PEG2 (S/AS), **5**/**6**) was evaluated by qPCR-based quantification of bcl-2 mRNA levels in MCF-7 cells. The indicated siRNAs were either transfected into the cells with lipofectamine 2000 (A), or applied without any uptake-enhancing agent (B). Cells were lysed after 24 h, total RNA was extracted, transcribed into cDNA, and quantified with qPCR. Bcl-2 levels were normalized to the reference gene RNA polymerase II, which was shown not to be influenced by siRNA or the transfection procedure. After lipofectamine-mediated delivery, efficient gene silencing occurred with both modified and unmodified siRNAs (*p* < 0.05 compared to untreated cells) with virtually no difference between PEGylated (**1**/**6** and **5**/**6**) and non-PEGylated siRNA (**1**/**2**) duplexes. In contrast, no gene silencing was detected when either of the siRNAs were applied without transfection enhancing agent, indicating no unassisted (gymnotic) uptake takes place. All values are reported relative to untreated cells (*n* = 3), error bars represent the standard deviation.

To confirm the results of the reporter assay, the siRNA PEGylated at either the sense (**1**+**6**) or both strands (**5**+**6**) were transfected into MCF-7 cells and the levels of endogenous bcl-2 were quantified by RT-qPCR. In correlation to the reporter assay, the attachment of short PEG chains had no detrimental effect of gene silencing activity (Fig. 5A). No statistically significant differences were detected between siRNAs with one PEG molecules at the sense or two PEG chains at both strands. After lipofectamine mediated transfection in a 1 nM concentration, the bcl-2 levels were reduced to 7–10%, and with 10 nM to 2–4% relative to untreated control samples.

For examination of a cell membrane permeation enhancing effect of the PEG-siRNAs, we also applied them in absence of any transfection agent. However, bcl-2 mRNA levels were unaltered (Fig. 5B), indicating that no unassisted uptake of these conjugates takes place.

**Figure 6:**
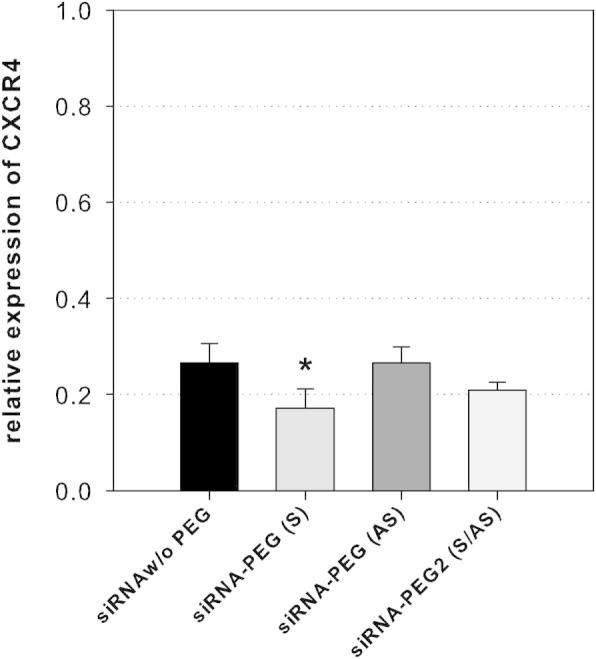
Gene knockdown of endogenous CXCR4. The effect of PEG-siRNA conjugates (siRNA-PEG (S), **7**/**10**; siRNA-PEG (AS), **9**/**8**; siRNA-PEG2 (S/AS), **9**/**10**) was evaluated by qPCR-based quantification of CXCR4 mRNA levels in MCF-7 cells. The indicated siRNAs were transfected into the cells at a concentration of 2 nM with lipofectamine 2000. Cells were lysed after 24 h, total RNA was extracted, transcribed into cDNA, and quantified with qPCR. All compounds resulted in efficient reduction of mRNA levels compared to untreated cells (*p* < 0.05, *n* = 3). When only the sense strand was PEGylated, statistically significant enhancement of the gene silencing effect was detected. Error bars represent the standard deviation, ^∗^ denotes *p* < 0.05 compared to cells treated with the unmodified siRNA.

The bcl-2 siRNA is highly active already in its unmodified state, and results in a 10-fold reduction of the corresponding mRNA. We hypothesized that the effect of PEGylation may be higher in cases where the down regulation effect of unmodified siRNA is only modest. To this end, we prepared a PEG conjugate of an siRNA targeted at CXCR4, a CXC chemokine receptor specific for stromal cell-derived factor-1.^23^ The wild-type siRNA results in a modest reduction of the CXCR4 mRNA to 27% when applied at a concentration of 2 nM. The siRNA duplex with only the antisense strand PEGylated showed nearly identical activity (Fig. 6). When both strands were modified, the targeted mRNA level was reduced to 21%, and with only the sense strand PEGylated, the effect was increased and resulted in a reduction to 17%, a statistically significant higher activity compared to the unmodified compound.

## 3 Discussion

Previously reported PEGylation strategies of antisense, aptamer or siRNA oligonucleotides relied on long linear or branched polyethylene glycols. The primary purpose of PEGylation is to achieve an increase in circulation time by reduction of renal filtration, and masking of immunostimulatory effects of modified oligonucleotides.^17^ In the case of pegaptanib, two 20 kDa PEG chains are attached to the aptamer to ensure retention at the intravitreal injection site.^24,25^ Nearly all reports of PEGylated antisense oligonucleotides used long PEG chains, usually in the range of 20–40 kDa, equivalent of around 450–900 ethylene glycol monomers.^26,27^ As an example, the PEGylation of oblimersen, a 20mer phosphorothioate antisense agent, resulted in a strong reduction of in vitro uptake in combination with lipofectamine transfection, but did not diminish the in vivo potency.^27^ This discrepancy was explained by a constant release of the active antisense agent by cleavage of the PEG chain.

For siRNA applications, PEGylation of siRNA at the 3′- or 5′-position has been reported with polydisperse PEG chains of an average molecular weight between 5 and 20 kDa.^19–21^ One study reported a decrease in silencing efficiency of a VEGF-targeted oligonucleotide compared to naked siRNA when PEG 5k was attached using a non-cleavable maleimide linker.^20^ In contrast, the application of a disulfide tether, which is expected to be cleaved in the endosome after successful uptake, resulted in minimized reduction of the gene silencing effect. PEGylation was tolerated on 3′- and 5′-ends of both strands, and after transfection with standard reagents, the extent of target down regulation was slightly lower to that achieved with unmodified siRNA. In a recent study, PEG with average molecular weights of 5, 10, and 20 kDa were attached to an LNA-modified siRNA at the 5′-end of the sense strand via click chemistry.^21^ Knockdown efficiencies of 50–60% were found for PEGylated siRNA, while the unmodified compound was considerably more effective with 90% reduction of the target gene. In both cases, the reduced silencing activity was attributed not to decreased cellular uptake, but rather to interference with the interaction of siRNA to the modulating RNAi enzymes dicer and RISC.^20^ The reduction in potency was proportional to the length of the PEG chains.^21^ The main virtue of PEGylation is the prolongation of circulation time by reducing the renal filtration rates, and the 20 kDa PEG-siRNA conjugate showed an increase in blood half-live from 5 min to 1 h.^21^

Consequently, we aimed to evaluate the gene silencing activity of conjugates of siRNA with short PEG ligands. We conjugated monodisperse Me-(PEG)_12_, affording the generation of a molecule with defined structure and molecular weight. PEGylation with high molecular weight ligands is only possible with a complex mixture of PEG chains of different length, yielding polydisperse conjugates, which in turn complicates chromatographic and mass spectrometry analyses as well as biological evaluations.^28^ The reaction of NHS–PEG with amino-modified single stranded RNA oligonucleotides afforded nearly complete conversion of the oligonucleotide after 3 h at room temperature in a borate buffer as monitored by HPLC. Ligand attachment at the siRNA is commonly preferred at the sense strand, because of minor interference with RISC strand recruitment and mRNA cleavage.^16^ Small molecule ligands attached at the 5′-end of the antisense strand have been shown to cause detrimental effects on gene silencing activity. The attachment of the amino linker at the 3′-position was achieved by the use of a commercially available modified CPG resin.^22^ However, the cleavage and deprotection protocol had to be modified to ensure complete deprotection of the amino group. The conditions recommended from the manufacturer proved to be insufficient for cleavage of the nitrophenyl masking group, and the respective impurity, the identity of which was confirmed by ESI-MS, was detected in up to 50% amount in HPLC traces.

After successful conjugation, the PEGylated RNA oligonucleotides were easily purified by semi-preparative RP-HPLC, taking advantage of the higher retention time of the PEGylated product. Initial evaluation of the gene silencing potency was performed with a luciferase reporter assay. To be able to monitor the activity of the bcl-2 targeted siRNA, we generated a psiCHECK2 plasmid with the bcl-2 cDNA inserted before the stop codon of Renilla luciferase. The luciferase reporter assay showed significant and potent bcl-2 down regulation by PEG-siRNA conjugates with standard lipofectamine transfection. Remarkably, no statistically significant differences resulted between siRNA PEGylated at either the sense or both sense and antisense strand, and the unmodified oligonucleotide. The shorter PEG chain is obviously better tolerated by the RNAi effector molecules than PEG 5 or 10 kDa,^20,21^ which resulted in reduced silencing potency.

The effects on the endogenous bcl-2 levels were investigated by qPCR analyses after treatment of MCF-7 cells with PEGylated siRNA in the presence and absence of a transfection enhancer. Confirming the results generated with the luciferase reporter system, we found a potent mRNA knockdown for two different gene targets. PEGylation of either the sense or both strands did not have any negative influence on the effect, proving the compatibility of short PEG with the RNAi effectors. Encountered differences of gene silencing potency between unmodified and PEGylated siRNA were slim and dependent on the gene target. Statistically significant enhancements through the PEG chains were only found in a single case: When using a duplex of PEGylated sense and unmodified antisense (**10**+**8**) targeted at CXCR4, the knock down activity increased. The CXCR4 gene is a chemokine receptor and has recently attracted attention as a prognostic factor for disease relapse and survival in leukemia patients. It is involved in tumor chemoresistance and a target for increasing chemosensitivity. In contrast to the highly active anti-bcl-2 siRNA (**1**+**2**), the wild-type CRCX4-targeted sequence (**7**+**8**) is only moderately efficient with a depletion of the corresponding mRNA of less than 74%. This value leaves some room for improvement, and the sense conjugate duplex (**7**+**10**) showed enhanced potency (83% mRNA reduction). A similar effect is arguably inherently impossible for those sequences that are already highly efficient in their unmodified form, as is the case for the bcl-2 targeted siRNA **1**+**2**.

No silencing effect was detected when the conjugates were applied without lipoplexes. In some cases, an increase in cellular uptake by PEGylation of oligonucleotides with long-chain PEG was reported,^26,29^ but others have shown equal^21^ or even poorer^27^ membrane permeation. The uptake seems to be dependent on cell type, and PEGylated oligonucleotides (having a large molecular weight) are supposedly being taken up by macropinocytosis. Cellular uptake and particularly endosomal escape is regarded as being pivotal for successful siRNA therapy, but no chemical modification or ligand conjugation has so far convincingly improved these properties. The finding that the conjugates are active siRNA agents and in some cases enhances their potency is encouraging for further development and combination with liposomal or nanoparticulate delivery systems.

## 4 Conclusion

Although the PEG-siRNA conjugates are arguably too small to support a significant reduction in plasma clearance, the finding that the activity is retained and in some cases even enhanced is encouraging. The conjugation protocol is fully compatible with other chemical modifications for increasing stability, such as 2′-methylation and phosphorothioate backbones. The short PEG chain may serve as a handle for interaction with PEGylated delivery systems, including lipoplexes and nanoparticles.^18^ In addition, the attachment of targeting molecules thorough a PEG spacer is a feasible possibility for improving the pharmacokinetic parameters, because the conjugation chemistry has no effect on the pharmacologic potency.

## 5 Materials and methods

### 5.1 Oligonucleotide synthesis

Synthesis of the anti-Bcl-2-siRNA was performed on the PolyGen 10 column DNA/RNA synthesiser (Polygen) using standard protocols with 15 min coupling time, 2′-*O*-*tert*-butyl-trimethylsilyl (TBDMS) nucleoside phosphoramidites and dicyanoimidazol (DCI) activator. For attaching an amino linker at the 3′ terminus, amino-on-CPG was used, and the first nucleotide was coupled on the synthesizer. All oligonucleotide synthesis reagents were purchased from SAFC-Proligo (Hamburg, Germany).

After the oligonucleotide assembly, the solid support was transferred into a vial and incubated with 1 ml of AMA (methanolic methylamine/NH4OH; 1:1) for 10 min at 65 °C. Because of incomplete deprotection of the aminohexyl linker, the cleavage conditions were later changed to reaction with ammonia for 4 h at 55 °C*.* The supernatant was collected in a sterile tube. The support was rinsed twice with 100 μl of RNase-free water. Subsequently the samples were dried under vacuum (Christ Speed-Vac RVC-2-18). The dried oligonucleotides were dissolved in 50 μl anhydrous DMSO (dimethylsulfoxide), 50 μl of TEA∗3 HF were added and the mixture was heated to 65 °C for 2.5 h.

Oligonucleotides were precipitated by addition of 200 μl sodium acetate (0.3 M) and 1 ml butanol. After incubation for 20 min −70 °C, the samples were centrifuged for 10 min at 12,500 rpm, the butanol was decanted and the pellet was washed twice in 750 μl cold 75% ethanol. The supernatant was again decanted and the dried pellet was dissolved in 50 μl RNase free water for absorption measurement (Nanodrop-1000, Thermo Fisher Scientific, Waltham, MA, USA). The concentration of siRNA was calculated according to the Lambert–Beer law with extinction coefficients calculated from nearest neighbor values.^30^ The oligonucleotides were diluted to a concentration of 100 μM and stored at −80 °C. siRNA duplexes were obtained by heating equivalent amounts of antisense and sense strands to 65 °C for 3 min followed by cooling down to room temperature.

### 5.2 Conjugation of siRNA with polyethylene glycol

A solution of single stranded RNA oligonucleotide (20 nM) was diluted to 100 μl in 100 mM sodium borate buffer (pH 8.0). 4 μl of a 250 mM solution of Me(PEG)_12_–NHS (Pierce, Thermo Scientific, Rockford, IL, USA) stock solution in DMF was added. The mixture was stirred for 1 h at room temperature and the reaction was monitored with HPLC or gel electrophoresis.

### 5.3 Polyacrylamide gel electrophoresis

One nanomole of each sample was diluted with 10 μl formamide loading buffer. The samples were heated for 3 min at 95 °C before being loaded onto the gel. The gel was run for 1.5 h at 150 V. For band visualization, the gel was agitated in a 2% solution of methylene blue for 30 min and subsequently destained with water. The gel was scanned with a densitometer (Bio-Rad GS-710, Carlsbad, CA, USA) and analyzed with Quantity One 4.6.3 1-D Analysis Software (Bio-Rad).

### 5.4 High performance liquid chromatography (HPLC)

All HPLC runs were performed with a LaChrom L-7100 (Merck Hitachi) system equipped with a Clarity 5 micron Oligo-RT 250 × 4.6 mm column (Phenomenex, Aschaffenburg, Germany) and the EZ Chrome Elite software. For analytical and semi-preparative runs, a linear gradient of 5–30% acetonitrile in triethylammonium acetate (TEAA) buffer in RNase-free water during 30 min was used at a flow rate of 1 ml/min. After a semi-preparative run, the fractions containing the separated oligonucleotides were dried, and diluted RNase free water.

### 5.5 Electrospray ionization mass spectrometry (ESI-MS)

Samples for mass spectrometry were desalted by ethanol precipitation from ammonium acetate.^31^ 30 μl of a 25 μM siRNA sample were mixed with 15 μl of a 7.5 M ammonium acetate solution and left at room temperature for 1 h. After adding 115 μl of ethanol the samples were left overnight at −80 °C. The samples were centrifuged for 30 min at 17,400×*g* and 0 °C to collect the precipitated oligonucleotides. The ethanol was decanted immediately after centrifugation and the remaining pellet was washed with 100 μl of ice cold 75% ethanol and centrifuged for 20 min. The supernatant was again decanted instantly, the pellet was air-dried and dissolved in 32 μl of RNase free water. For MS analyses, the samples were diluted in acetonitrile and triethylamine to obtain a final ratio of 50:50:1 (water/ACN/TEA). Analysis was performed on a micrOTOF-Q II 10240 (Bruker, Billerica, MA, USA). Calibration of the system was carried out with lithium-formate. The samples were measured in negative ion mode.

### 5.6 psiCHECK-2 Bcl-2 plasmid

The psiCHECK-2-vector, obtained from Promega GmbH (Madison, WI, USA), was digested with EcoRI (Fermentas, Thermo Scientific, Waltham, MA, USA), and converted to a Gateway® destination vector using a Gateway® Vector Conversion System (Invitrogen, Life Technologies, Paisley, UK) by ligating the reading frame cassette according to the manufacturer’s instructions. The resulting destination vector was sequenced to confirm correct orientation of the reading frame cassette. The human bcl-2 ORF shuttle expression clone with a Gateway® entry vector was obtained from ImaGenes (Source BioScience, Berlin, Germany). The psiCHECK2-bcl-2 plasmid was generated by an LR recombination reaction and transformed into *Escherichia* *coli* XL1-Blue. The vector was sequenced to confirm successful cloning, and isolated from *E. coli* XL1-Blue using a GeneJET™ Plasmid Miniprep Kit (Fermentas, Thermo Scientific).

200 μl of an *E. coli* XL-1 Blue stock were transformed by heat-shock with 100 ng psiCHECK-2-bcl-2 plasmid and plated onto an ampicillin agar-plate. Miniprep cultures were grown from a single colony. The plasmid was isolated with the GeneJET Plasmid Miniprep Kit (Fermentas, Thermo Scientific) according to the manufacturer’s instructions.

### 5.7 Cell cultivation and seeding

Cells were cultivated in Dulbecco’s Modified Eagle Medium (DMEM) with GlutaMAX™ (Gibco, Life Technologies, Carlsbad, CA, USA) supplemented with 10% FBS (PAA, Pasching, Austria) at 37 °C in 5% CO_2_. The cell line MCF-7 was obtained from the European cell culture collection (EACC). Cells were trypsinized and resuspended in an appropriate volume of preheated culture medium, and counted with a Neubauer chamber. 3 × 10^4^ cells were plated in 96-well plates for the luciferase assays, and 1 × 10^5^ cells were used in a 24 well plates for qPCR assays. Each treatment was performed in triplicate.

### 5.8 Dual-luciferase reporter assay

For reverse co-transfections, a mix of including the psiCHECK2-bcl-2 plasmid (100 ng/well), the siRNA 0.1–10 pMol/well) and Lipofectamine™2000 (0.25 μl/well) was prepared in Opti-MEM (50 μl/well) according to the manufacturer’s instructions. The transfection mixture was pipetted into the multiwell plate and incubated at room temperature for 20 min before the cell suspension (100 μl per well) was added. Cells were incubated for 48 h.

For the assessment of unassisted uptake of the conjugates, a mixture of the psiCHECK2-bcl-2 plasmid (100 ng/well), and Lipofectamine™2000 (0.25 μl/well) was prepared in Opti-MEM (50 μl/well) according to the manufacturer’s instructions. The transfection mixture was pipette into the multiwell plate and incubated at room temperature for 20 min before the cell suspension was added. After 24 h of incubation the medium was removed, and the indicated amount of siRNA diluted in 100 μl DMEM with 10% FCS was transferred to each well. Cells were incubated for a further 48 h.

For the luminescence-based quantification of gene knockdown, the Dual Luciferase Assay (Promega, Madison, WI, USA) was used according to the manufacturer’s instructions. The cell medium was removed, cells were washed with PBS, and lysed for 20 min at rt with 20 μl lysis buffer per well. The substrates were dispensed using the plate reader’s (Tecan infinite M200Pro) injector and the luminescence was measured immediately. For normalization of transfection efficiency and cell growth, the ratio of renilla and firefly luciferase was calculated.

### 5.9 RNA extraction and cDNA synthesis

After 48 h incubation the whole RNA was extracted. Column based RNA extraction was performed according to the GeneJET RNA Purification Kit (Fermentas, Thermo Scientific) manufacturer manual. The concentration and purity of the extracted RNA was determined with a Nanodrop spectrophotometer. Five-hundred nanograms total RNA of each sample were used for cDNA synthesis with the RevertAid First Strand cDNA Synthesis Kit (Fermentas, Thermo Scientific) with a random hexamer primer in an Eppendorf Thermo Cycler according to the manufacturer’s instructions. Two microliter of 1:10 diluted cDNA were used for each qPCR reaction. The master mix was prepared with Hot FirePol Polymerase and EvaGreen from Solis Biodyne (Tartu, Estonia) and gene-specific primers for bcl-2 (forward: CCCAAGTTTTGAGCCATTCA, reverse: CCTGGTGGACAACATCGC), CXCR4 (forward: CGTGGAACGTTTTTCCTGTT, reverse: GGTGCTGAAATCAACCCACT), and RNA polymerase II (forward: GAAGGCACTCTCCAGGTTTG, reverse: ATGCTGGTTTTGGTGACGAC) as reference gene from Microsynth (Basel, Switzerland). Primer design was done in compliance with the Miqe guidelines^32^ using the online tools Primer3Plus, mFOLD (amplicon total Δ*G* ⩾ −3.0; no secondary structures at the primer binding side), NetPrimer (no hairpins; dimer Δ*G* ⩾ −6.0; cross dimer Δ*G* ⩾ −6.0), and PrimerBlast; see Supplementary information. Standard curves for determination of the reaction efficiencies were prepared by making serial dilutions of the untreated reference cDNA. PCR reactions were run on a Roche Light Cycler 480 (Roche, Mannheim, Germany) with two technical replicates for each sample. Cp values and reaction efficiencies (bcl-2: 0.864, CXCR4: 0.946, RNA polymerase II: 1.078) were determined using the Light Cycler software. The relative expression of bcl-2 normalized to the reference gene and the standard errors were calculated with REST 2009 software (Qiagen, Hilden, Germany).

## Figures and Tables

**Table 1:** Sequences and ESI-MS data of bcl-2 (**1**–**6**) and CXCR4 (**7** and **8**) targeted siRNA oligonucleotides

	Oligonucleotide sequence	Strand	Modification	Mass calcd	Mass found
**1**	UCA-GGU-ACU-CAG-UCA-UCC-ACA-dTdT	AS	—	7224.14	7224.03
**2**	UGU-GGA-UGA-CUG-AGU-ACC-UGA-dTdT	S	—	7361.22	7362.16
**3**	UCA-GGU-ACU-CAG-UCA-UCC-ACA-dTdT	AS	3′-Amino	7403.21	7403.26
**4**	UGU-GGA-UGA-CUG-AGU-ACC-UGA-dTdT	S	3′-Amino	7540.29	7540.15
**5**	UCA-GGU-ACU-CAG-UCA-UCC-ACA-dTdT	AS	3′-PEG	7974.54	7974.14
**6**	UGU-GGA-UGA-CUG-AGU-ACC-UGA-dTdT	S	3′-PEG	8111.62	8110.93
**7**	AAA-CUC-ACA-CCC-UUG-CUU-GdTdT	AS	—		
**8**	CAA-GCA-AGG-GUG-UGA-GUU-UdTdT	S	—		
**9**	AAA-CUC-ACA-CCC-UUG-CUU-GdTdT	AS	3′-PEG		
**10**	CAA-GCA-AGG-GUG-UGA-GUU-UdTdT	S	3′-PEG		

## Supplementary data

Supplementary data qPCR information and calculation of PCR efficiency.
