# Supplementary material for: Chemically defined polyethylene glycol siRNA conjugates with enhanced gene silencing effect
Source: Bioorg Med Chem. 2014 Apr 1;22(7):2320–6. doi: 10.1016/j.bmc.2014.02.004 (PMC3994277; doi:10.1016/j.bmc.2014.02.004)
Supplement: Supplementary data — qPCR information and calculation of PCR efficiency. [file mmc1.pdf]

Chemically defined polyethylene glycol siRNA conjugates with enhanced gene silencing effect

Zuzana Gažiová , Volker Baumann, Anna-Maria Winkler and Johannes Winkler

qPCR information

gene name	*NCBI Acc. Nr.*	*amplicon length (bp, verified on agarose gel)*	*melting temperature (Tm) °C*	*qPCR efficiency*	*slope*	*Y intercept*	*for/rev*	*Sequence 5´ - 3´*	*exon spanning*
B-cell CLL/lymphoma 2 (BCL2)	NM_000633	146	92.07	0.864	-3.794	43.20	for	CCTGGTGGACAACATCGC	one exon, no introns
							rev	GAGACAGCCAGGAGAAATCA	
Chemokine (C-X-C motif) receptor 4 (CXCR4)	NM_001008540	125	85.45	0.946	-3.680	38.90	for	CGTGGAACGTTTTTCCTGTT	one exon, no introns
							rev	GGTGCTGAAATCAACCCACT	
Polymerase (RNA) II (DNA directed) polypeptide A, 220kDa (POLR2A)	NM_000937	133	87.34	1.078	-3.439	34.55	For	ATGCTGGTTTTGGTGACGACT	21 -22
							Rev	TCCATCTTGTCCACCACCTCT	

Calculation of PCR efficiency

Gene name	cDNA (2 µl 1:10 dilution)	1:80 dilution	1:640 dilution	1:5120 dilution	1:40980 dilution	RT- (2 µl 1:10 diluted cDNA)	RT- control in % from RT+, limit < 1.5%	No template control (NTC)	NTC in % from RT+, limit < 1.5%
B-cell CLL/lymphoma 2 (BCL2)	29.88	33.10	36.39	40.25	43.00	41.81	0.026%	44.00	0.006%
Chemokine (C-X-C motif) receptor 4 (CXCR4)	27.01	30.08	33.10	36.42	n/a	33.10	1.473%	43.00	0.002%
Polymerase (RNA) II (DNA directed) polypeptide A, 220kDa (POLR2A)	23.36	26.29	29.29	31.84	n/a	32.18	0.254%	34.60	0.047%
